# Adherence of Healthcare Professionals to American Diabetes Association 2004 guidelines for the care of patients with type 2 diabetes at Peripheral Diabetes Clinics in Karachi, Pakistan

**DOI:** 10.12669/pjms.292.3149

**Published:** 2013-04

**Authors:** Farzana Muzaffar, Nimra Fatima, Asher Fawwad, Mussarat Riaz

**Affiliations:** 1 Farzana Muzaffar, M. Phil, Research Officer, Baqai Institute of Diabetology and Endocrinology, Baqai Medical University, Karachi, Pakistan.; 2 Nimra Fatima, B.E (Bio Engineering), Research Officer, Research Department, Baqai Institute of Diabetology and Endocrinology, Baqai Medical University, Karachi, Pakistan.; 3 Asher Fawwad, MBBS, M.phil,Assistant Professor, Research Department, Baqai Institute of Diabetology and Endocrinology, Baqai Medical University, Karachi, Pakistan.; 4 Mussarat Riaz, FCPS, Consultant Physician, Department of Medicine, Baqai Institute of Diabetology and Endocrinology, Baqai Medical University, Karachi, Pakistan.

**Keywords:** Peripheral Diabetes Clinics, ADA 2004 guidelines, Type 2 Diabetes, Healthcare professionals

## Abstract

***Objective:*** To observe the adherence of Healthcare Professionals to American Diabetes Association (ADA) 2004 guidelines for the care of patients with type 2 diabetes at Peripheral Diabetes Clinics (PDCs) in Karachi, Pakistan.

***Methodology:*** The study was conducted using a retrospective medical chart review of patients with type 2 diabetes at four PDCs in four townships of Karachi district from January 2005 to December 2006. Entire medical records of patients were evaluated for the evidence of documentation of testing and treatment.

***Results:*** Medical records of 691 patients (332 males and 359 females) with type 2 diabetes were reviewed. Mean age of the patients was 50.79 ± 10.75 years. Deficiencies were observed in most areas of diabetes care. Blood pressure was documented in 85.81% patients, whereas, serum creatinine, HbA1c and lipid profile were noted in 56%, 44.57% and 40.08% of the patients respectively. Similarly, lower leg examination was registered in 44% patients, while in 30.53% of the patients fundoscopic examination was recorded. Co-morbid conditions like hypertension and hyperlipidemia were documented in 92.7% and 84.6% patients respectively. HbA1c < 7% was achieved by 59.04% patients, while 27.50% of the patients attained the recommended level of serum cholesterol. Likewise, ADA recommended goal for blood pressure and LDL was achieved by13.02% and 12.16% patients respectively.

***Conclusions:*** The study showed that adherence of healthcare professionals to ADA guidelines was suboptimal. Moreover, insufficient documentation of medical records reflected inadequate care of patients with type 2 diabetes.

## INTRODUCTION

Diabetes mellitus is one of the major threats to human health in the current century.^1^ It is mounting rapidly, spanning in wide populations and multiple ethnic groups of the world. According to the recent estimates, diabetes caused 4.6 million deaths worldwide in the year 2011.^2^ The global figure of people with diabetes is anticipated to rise from 366 million in 2011 to 552 million by the year 2030.^2^

Continuous medical care and patient education for self-management of diabetes is crucial, not only to prevent acute complications, but also, to reduce the risk of long-term complications of the disease.^3 ^Physicians often advise patients with diabetes to attain good glycemic control with adherence to physical activity and dietary guidance. Every year, American Diabetes Association (ADA) issues Clinical Practice Recommendations for diagnosis, treatment and prevention of diabetes. Furthermore, different countries also publish their own guidelines for the management of diabetes, in accordance with ADA and International Diabetes Federation (IDF) recommendations to help healthcare providers treat patients with diabetes using the most current research available.^4^

A number of studies assessed compliance of healthcare professionals to ADA guidelines in their clinical setting for the management of diabetes.^5^^-^^7^ A study reported association between adherence of primary care physicians to guidelines for diabetes and patient satisfaction.^8^

Hence, the present study was designed with the aim to assess the adherence of healthcare professionals to the ADA 2004 guidelines at peripheral diabetes clinics in Karachi Pakistan. 

## METHODOLOGY

***Study Population: ***A retrospective study was conducted from January 2005 to December 2006 at four peripheral diabetes clinics (PDCs), located in four different townships of Karachi, Pakistan. The clinics were chosen to reflect diversity in demography and socio-economic status of the local population. Ethical approval for the study was obtained from the Institutional Review Board (I.R.B) of Baqai Institute of Diabetology and Endocrinology (BIDE).

***Inclusion and Exclusion Criteria: ***The general population with diabetes, aged 20 years or older, living in middle and low socio-economic urban areas of Karachi were enrolled in the study. Pregnant females, patients aged less than 20 years and patients with type 1 diabetes were excluded from the study.

***Healthcare professionals: ***In order to achieve optimal care for patients with diabetes, a program was initiated to train physicians as Associate Diabetologists (ADs) with a view to support the Peripheral Diabetes Clinics (PDCs) and improve quality of care of patients with diabetes. The ADs practicing at PDCs were included in the study.

Medical records of the included patients were reviewed and evaluated for the evidence of documentation of testing and treatment in order to assess the status of primary care at peripheral diabetes clinics. Data collected from the medical records included demographic information, height, weight, body mass index (BMI), family history of diabetes, history of tobacco consumption, measures of glycemic control such as fasting blood sugar (FBS), random blood sugar (RBS) and glycosylated hemoglobin A1c, systolic blood pressure (SBP), diastolic blood pressure (DBP), fasting lipid profile, urine detailed report especially for albumin, microalbuminuria, 24 hrs urinary protein, 24 hrs creatinine clearance, serum creatinine, documentation or referral for dilated eye examination and lower limb assessment (pulses, reflexes, touch and vibrations). Diagnosis and treatment of chronic co-morbid conditions such as hypertension, dyslipidemia was also recorded.

**Table-I:** Baseline Characteristics of patients with type 2 diabetes at Peripheral Diabetes Clinics (PDCs

*Variables*	*Male*	*Female*	*p-value*	*Overall*
*n (%)*	*332*	*359*		*691*
Age (yrs)	51.40 ± 11.45	50.23 ± 10.05	0.157	50.79 ± 10.75
Duration of Diabetes (yrs)	10.66 ± 6.52	11.47 ± 6.16	0.100	11.09 ± 6.34
Weight (kg)	72.08 ± 12.49	65.68 ± 13.78	0.000	68.81 ± 13.54
Height (cm)	167.02 ± 7.34	154.26 ± 7.10	0.000	160.61 ± 9.63
Body Mass Index (kg/m^2^)	25.85 ± 4.16	27.53 ± 5.18	0.000	26.69 ± 4.77
Systolic Blood Pressure (mmHg)	136.42 ± 20.14	139.66 ± 19.52	0.047	138.10 ± 19.87
Diastolic Blood Pressure (mmHg)	84.84 ± 9.98	86.27 ± 9.32	0.072	85.58 ± 9.66
Fasting Blood Glucose (mg/dl)	192.60 ± 69.00	196.03 ± 72.28	0.642	194.32 ± 70.59
Random Blood Glucose (mg/dl)	276.65 ± 102.46	280.97 ± 99.24	0.620	278.86±100.75
HbA1c (%)	9.12 ± 1.66	9.15 ± 1.60	0.885	9.13±1.63
Serum Creatinine (mg/dl)	1.0 ± 0.27	1.05 ± 0.61	0.349	1.02±0.48
Total Cholesterol (mg/dl)	194.04 ± 40.09	194.26 ± 45.34	0.965	194.15±42.79
Triglyceride (mg/dl)	231.84 ± 115.82	216.33 ± 120.22	0.259	224.0 ± 118.12
High Density Lipoprotein (mg/dl)	38.41 ± 7.56	39.86 ± 6.59	0.082	39.16 ± 7.10
Low Density Lipoprotein (mg/dl)	118.89 ± 31.01	116.46 ± 31.35	0.505	117.62 ± 31.62

Data presented as Mean ± SD p < 0.05 considered statistically significant

**Table-II:** Documented demographic, anthropometric and clinical variables of patients with type 2 diabetes at Peripheral Diabetes Clinics

*Demographic Variables*	*Documented*
Age (years)	98.55 %
Duration of diabetes (years)	96.1 %
Family history of Diabetes (years)	98.40 %
Tobacco consumption	96.96 %
*Anthropometric Variables*	*Documented*
Weight (kg)	89.86 %
Height (cm)	84.94 %
Body Mass Index (kg/m^2^)	84.66 %
*Clinical Variables*	*Documented*
Fundoscopy	30.53 %
Lower Leg Examination	44.0 %
Foot Ulcer	2.02 %

Data presented as %

**Table-III:** Documented ADA standards of treatment in patients with type 2 diabetes at Peripheral Diabetes Clinics

*ADA standards of treatment*	*Documented*
Fasting Blood Glucose (mg/dl)	53.25%
Random Blood Glucose (mg/dl)	77.42%
HbA1c (%)	44.57%
Total Cholesterol (mg/dl)	46.16%
Triglyceride (mg/dl)	42.98%
High Density Lipoprotein (mg/dl)	42.11%
Low Density Lipoprotein (mg/dl)	42.69%
Serum Creatinine (mg/dl)	56.0%
Urine Analysis	27.78%
Microalbuminuria (mg/dl)	24.89%
Blood Pressure (mmHg)	85.81%

Data presented as %

**Fig.1:**
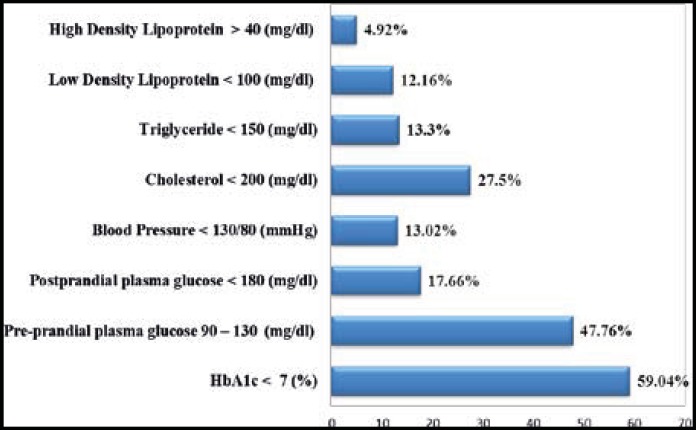
Compliance of patients with type 2 diabetes to ADA recommended goals of treatment

All related variables found on the medical records were used to assess the compliance of healthcare professionals to the recommended ADA 2004 guidelines for the management of type 2 diabetes. Recommended ADA 2004 guidelines are summarized as above:

***Statistical analysis: ***The data was analyzed on Statistical Package for Social Sciences (SPSS) version 13.0. Demographic, anthropometric and biochemical results presented as Mean ± SD and percentage. t- test was employed to compare the characteristics of males and females. p<0.05 was considered statistically significant.

## RESULTS

Table-I shows mean anthropometric, clinical and biochemical variables of patients with type 2 diabetes at each peripheral diabetes clinic. Medical records of 691 patients with type 2 diabetes (332 males and 359 females) were assessed for evaluating adherence of healthcare professionals to ADA recommended guidelines. Mean age of males was 51.40 ± 11.45 years and that of females was 50.23 ± 10.05 years. Mean duration of diabetes was 11.09 ± 6.34 years and mean HbA1c was 9.13 ± 1.63 %. Similarly, mean SBP and DBP was 138.10 ± 19.87 and 85.58 ± 9.66 mmHg respectively. About half of the study population was found obese (BMI ≥ 25kg/m^2^); among them females were more obese compared to males (p=0.000).

Details of documented demographic, anthropometric and clinical variables of 691 patients with type 2 diabetes at all Peripheral Diabetes Clinics are shown in Table-II. Of the entire medical record, demographic information of patients was the most documented variable at PDCs. Weight was registered in 89.86% patients while 84.94% and 84.66% of the patients had their height and BMI recorded respectively. Of the clinical variables, lower leg examination was documented in 44% patients, eye examination in 30.53%, whereas, foot ulcer examination was recorded in 2.02% of the patients.

Documentation of lower leg examination indicated that popliteal pulses were observed in 45.87% (n=317) patients; posterior tibialis and dorsalis pedis in 47.03% (n=325) patients while vibration sense and touch sensation was recorded in 42.83% (n=296) and 42.98% (n=297) patients respectively. Knee jerk reflex was documented in 42.54% (n=294) patients while ankle jerk reflex was recorded in 42.98% (n=297) patients.

Likewise, blood pressure of 85.81% (n=593) patients was recorded at their initial visit to PDCs. Out of the measures of glycaemic control; fasting blood sugar was recorded in 53.25% patients, random blood sugar in 77.42% while HbA1c levels were noted in 44.57% of the patients. Serum cholesterol was reported in 46.16% (n=319) patients and triglycerides in 42.98% (n=297) whereas 42.11% (n=291) patients had their HDL levels documented as shown in Table-III.

Similarly, lipid profile was documented in 40.08% (n=277) patients. HDL (High Density Lipoprotein) level ≤ 40 mg/dl was observed in 36.76% (n=107) males while 145 (49.82%) females had HDL level ≤ 50 mg/dl.

Hypertension (BP >130/80 mmHg) was seen in 92.7% patients; more in females with 27% patients taking anti-hypertensive medicine. Likewise, hyperlipidemia was observed in 84.6% patients with 15.6% of the patients on lipid lowering medication.

Fig.1 shows proportion of the study population, meeting the ADA goal of treatment. The ADA recommended goal of HbA1c was achieved by 59.04% patients while 47.76% and 27.50% of the patients attained the recommended level of FBS and serum cholesterol respectively. Likewise, ADA goal for RBS, blood pressure and LDL (Low Density Lipoprotein) was reached by 17.66%, 13.02% and 12.16% of the patients respectively.

## DISCUSSION

This study indicates that ADA guidelines were not being completely implemented at PDCs. The patients with type 2 diabetes had poor glycemic control and high prevalence of co- morbidities. Screening of clinical variables in patients with type 2 diabetes was not good, especially for eye, microalbuminuria and lower extremities whereas documentation of anthropometric measurements and blood pressure was relatively better.

Glycated haemoglobin (HbA1c) is a strong indicator of glycemic control.^10^ The mean value of HbA1c found in our study was slightly higher than those observed in Bangladesh, Malaysia and closer to that found in India.^11^^-^^13^ More than three-fourth (90%) of the study population had poor glycemic control on their first visit to the PDCs. This finding indicates high susceptibility of patients with type 2 diabetes to develop early diabetic complications due to poor glycemic control.^10^ Moreover, it also indicated that patients coming to PDCs did not have their diabetes under optimal control.

In our study, about 40% of the patients had HbA1c and lipid profile documented. Moreover, a large proportion of the study population did not achieve ADA recommended standards of treatment. Similarly, a study conducted in Thai population indicated that HbA1c and lipid profile was documented in more than half of the study population, while blood pressure was recorded in all the patients. ADA goal of treatment was attained by 49% for HbA1c and 64% for LDL and HDL; however only a small proportion reached ADA goal of blood pressure.^14^

Likewise, while evaluating adherence to ADA guidelines in endocrinology and primary-care clinic, significant differences were seen. Endocrinology clinic was better in majority of the clinical and biochemical areas. Significantly lower values of HbA1c and fairly good documentation of lipid profile and foot examination was observed in endocrinology clinic relative to primary-care set-up.^15^

Dyslipidemia, prevalent in patients with type 2 diabetes, increases risk for atherosclerotic vascular disease.^16^^,^^17^ In our study, overall undesirable lipid profiles were seen. Significantly high triglycerides and low HDL, typical of diabetes dyslipidemia were also seen in a study conducted in India.^18^ It has been observed that effective management of lipid measurement reduces secondary vascular events.^16^^,^^17^

Although majority of the patients in the study had their blood pressure checked; only one fourth were taking anti-hypertensive medications. However, the overall result indicates that family physicians were better at documenting blood pressure than lipid profile. A study conducted in Karachi also showed similar suboptimal standards of diabetes care by the healthcare providers.^19^

ADA recommends that dilated eye examination (fundoscopy) should be performed shortly after diagnosis of diabetes and then annually. In our study a smaller proportion of patients had eye examination at their first visit. Since we did not audit the medical records of these patients for annual reviews; we may assume that some of these patients had their annual screening tests and examinations on their annual review visits.

A number of studies highlighted different barriers in the optimal management of diabetes like lack of skilled personnel in the health-care system, socio-economic status and lack of awareness of patients.^5^^,^^7^^,^^20^ Similarly, a study proposed improved adherence of health-care professionals to ADA after the implementation of necessary interventions.^7^

Limitations of the study like, lack of knowledge of current recommendations by the family physicians, patient compliance issues, including medication cost and side effects, lack of documentation of medical records and available diabetes educators need to be taken into consideration.

## CONCLUSION

Study findings showed that most of the medical records of patients were inadequately filled and lacked proper documentation. Screening evaluations, recommended by ADA guidelines for early detection of diabetes complications were suboptimal at all the PDCs. Furthermore, poor record maintenance, shown in our study, signify the importance of periodic auditing of medical records and forms at healthcare centers in order to assess and improve the quality of diabetes primary care given to the patients.

## Authors Contribution

**FM and AF: **Conception and design or acquisition of data or analysis and interpretation of data, drafting the article or revising it critically for important intellectual content and final approval of the version to be published.

**NF:** Conception and design or acquisition of data or analysis and interpretation of data and final approval of the version to be published.

**MR:** Drafting the article, revising it critically for important intellectual content and final approval of the version to be published.

## Notes

Note: This study was a part of M. Phil Thesis and its publication got delayed due to some personal reasons of the main investigator.
